# Foreign Correspondence

**Published:** 1863-09

**Authors:** 


### FOREIGN CORRESPONDENCE.

PARIS, July 31st, 1863.

Prof. E. INGALS, M. D.,—*My Dear Doctor*, The first impressions of Paris differ entirely from those obtained by a first look at London. We have left behind us the fog and mists, the fitful sunshine and blackened walls of that immense city; and are now in the genial atmosphere of unclouded skies, surrounded by the light and cheerful edifices of Paris. Probably no city ever underwent greater or more rapid changes in external appearance than this, during the last ten years. Changes, too, which contribute not only to the beauty, but also to the Sanitary condition of the city. If Gibbons could speak of Paris as "the splendid capital which now embraces an ample territory on either side of the Seine," what would he say of the Paris of 1863, could he behold the imposing grandeur and magnificence of her modern buildings—the sweep and breadth of her boulevards, squares and gardens which have displaced the high, gloomy and desolate looking houses, and narrow lanes and passages into which the fresh air never penetrated, and the sun's rays never shone. The scene that meets the eye in an external survey of the city, is in keeping with the beauty of architectural design—for all that appears upon the surface leads one to the conclusion that pleasure is the uninterrupted occupation of the people. One continued display of gayety and festivity is almost always and everywhere observed, to the apparent disregard of the natural divisions of time—they whirl along much the same through all the hours of day or night—and if perchance overtaken by fatigue, stop for a short time, and start again as before. Indeed a doubt at first rests upon the mind, whether in the midst of this excitement material could be found to render even a brief communication of any interest, but there are other scenes in this beautiful, gay and festive city. When permitted to look behind the screen, as the physician may do, other sights are beheld which extort sympathy from the most unwilling heart, and affords a saddening contrast to the glitter of gayety which the surface presents. We have but to walk through any of the numerous hospitals here to behold the wonderful transition which a short course of dissipation will effect, in the numerous victims gathered here from all conditions of life, written in the wan and faded cheek, the wrinkled brow, the palsied limb, and the anguish stricken heart.

The hospitals of Paris receive annually 90,000 patients, 5,-200 of whom are always under treatment, besides these the directors grant relief yearly to 50,000 indigent families. These institutions are supported in part from the public treasury—largely by private munificence—a heavy tax is levied on every piece of ground purchased for the purpose of burial in cemeteries, and also a tax of 8 per cent. on the receipts of places of amusement, to aid in defraying the expense of the hospitals of the city—this latter fact is suggestive of what should be done in our own country for the same purpose.

The number of physicians in this city is about 2,000, or one to every 750 of the inhabitants. The number of medical students is about 1,200, who are at present undergoing their examinations, it being near the close of the lecture term.

No professional man fails to visit the wards of the Hotel Dieu, as it is the most ancient institution of the kind in Paris—its foundation dating back so far as the year 660—and also because it may be taken as a model of all the others. It is composed of three detached portions connected by covered archways, and an aqueduct leading under the quay. It receives annually about 12,000 patients. On the walls of the vestibule are seen the portraits of Bichat, Deraust, Moreau, Dupuytren, Bonden, Mery, Desault, and Thebault. While standing within these ancient walls—in the very theatre of the brilliant career of so many worthies who have contributed to the advancement and elevation of medical science—one almost feels a solemn veneration for our noble profession.

We meet here on active duty M. Trousseau, DeMussy, Barth, Maisonneuve, and others.

The Hospital Lariboisiere is a modern and, in its design, a model institution, the general form is rectangular. A tasteful colonade fronts a spacious court, inclosed by nine uniform pavilions, which are separated by smaller courts and gardens, thus guarding against the extension of contagious diseases. Here we witness the operations of M. Chassaignac, many of which are performed with the ecrassum. From prejudice or some other cause this instrument has not obtained much popularity with surgeons here.

At LaCharité M. Velpeau is in active service, and followed by a large class of students.

The clinics of M. Paul Dubois are attended by many students who are just completing the period of their pupilage, and preparing for the duties of private practice.

The institution of not the least interest is the Musee Dupuytren, where the effects of almost every variety of disease are represented, and may be studied in some one or other of the numerous pathological preparations preserved here.

The physician can obtain a just idea of the "*Expectante*" mode of treating disease, only by walking the wards of the Parisian hospital. Here he will be impressed with the care and doubtless the accuracy in making diagnoses—and when that is reached, as if the only object was attained—the inevitable "*Potage*" is prescribed till the next visit—possibly some liniment may be applied externally. M.

PARIS, Aug. 12th, 1863.

In some respects the medical profession of France enjoys an enviable position. The stringent requisitions for entering its ranks, and the laws regulating the practice, at the same time protect the community and guard the interests of the fraternity. There are only three schools in France which may grant degrees conferring the right to practice in any part of the Empire. They are located, one each at Paris, Montpellier and Strasbourg. There are, however, Secondary Schools of Medicine in other parts of this country where the student may pursue his studies. Thus the profession is seldom brought into competition with the ignorant and vaunting mountebank. An incident occurred here within the last week which shows the mode of treating such cases when they occur. One "Dr. Vries," the "*Black Doctor*," who some time since obtained some notoriety in a similar way, was arrested on the charge of usurping the title of doctor, and of illegally practising medicine. The proof was deemed sufficient to convict, and the penalty was fixed at a fine of 2,000 francs and six months imprisonment, and he to pay costs of the prosecution.

The École et Maison D'Accouchement has 416 beds. Women are received here in the last month of their pregnancy, and are attended in their confinement by females, or in difficult cases by the surgeon of the establishment. The mean term of the patient's abode here is 18 days. Medical students are excluded from this Hospital, which is devoted to the instruction of young women as midwives. After a course of two years, these pupils are examined, and are allowed to practice on receiving a diploma. The number of licensed midwives in Paris is about 450.

M. Depaul, who succeeds M. Dubois to the chair of Obstetrics in the Faculty of Medicine, is spoken of in the highest terms both as a gentleman and practitioner. And notwithstanding he does not bring the prestige of the name of Dubois he is acceptable as a teacher. M. Pajot's *lectures* on the same subject are spoken of as very good.

Hopital Necker, situated in the southwest part of the city, contains 386 beds, 12 of which are appropriated to calculary diseases, and are under the control of M. Civiale· The operation of lithotomy, I understand, is seldom performed in this institution. Yet while the stone is broken down and removed from the bladder piecemeal, it is at the expense of so much time and local injury to the passage, that I am not surprised the operation of Lithotomy has not been generally adopted. One is sometimes disappointed in not seeing greater dexterity and brilliancy in the operative surgery of Parisian Hospitals.

Chloroform is considerably used here, but I am in doubt whether their patients receive all the benefit from it that they should. I have not witnessed a single operation of any importance in which the patient did not manifest the greatest suffering. M. Chassaignac, in his clinic, said very truly, that chloroform should be introduced into the system *slowly;* and when it began to affect the patient, if no unpleasant manifestations occurred, then it may be administered to almost any extent. Then I was at a loss to understand why sufficient was not given, if not to remove the pain of the operation, at least to assuage the suffering.

The "Jardin des Plants" is one of the most interesting points in the city. It is really a most perfect school for the study of the natural sciences. The collection of animals is very extensive. The Botanical garden covers a large extent of ground and is most admirably arranged. The Museum of Comparative Anatomy is said to be unequaled. In the department of Geology, Mineralogy, Materia Medica, &c., nothing is left to be desired. Free access is permitted to all visitors, whether for the purpose of study or curiosity.

Among the many enthusiastic cultivators of Medical Science here, few if any have been more industrious or successful than M. Ordonez, who is almost entirely devoted to Histology In his classes are many students from America, who are universally pleased with him. He is engaged, besides teaching, in the preparation of a systematic work, based on original experiments and observations, which with his peculiar arrangement and classification, promises to be a valuable addition to the literature upon that subject.

The weather has been uninterruptedly delightful in Paris for weeks past, but for the last four days the heat has been quite oppressive. The thermometer ranging from 95° to 97° in the middle of the day.

Unprecedented preparations are being made for the annual fete, on the 15th inst., which, it is determined, shall *outshine* all previous demonstrations of the kind. M.

